# Altered Effective Brain Connectivity During Habituation in First Episode Schizophrenia With Auditory Verbal Hallucinations: A Dichotic Listening EEG Study

**DOI:** 10.3389/fpsyt.2021.731387

**Published:** 2022-01-03

**Authors:** Leilei Zheng, Weizheng Yan, Linzhen Yu, Bin Gao, Shaohua Yu, Lili Chen, Xiaoyi Hao, Han Liu, Zheng Lin

**Affiliations:** ^1^Department of Psychiatry, Second Affiliated Hospital, School of Medicine, Zhejiang University, Hangzhou, China; ^2^Tri-institutional Center for Translational Research in Neuroimaging and Data Science (TReNDS), Georgia State University, Georgia Institute of Technology, Emory University, Atlanta, GA, United States

**Keywords:** schizophrenia, habituation, effective connectivity, dichotic listening, auditory verbal hallucination

## Abstract

**Background:** Habituation is considered to have protective and filtering mechanisms. The present study is aim to find the casual relationship and mechanisms of excitatory–inhibitory (E/I) dysfunctions in schizophrenia (SCZ) via habituation.

**Methods:** A dichotic listening paradigm was performed with simultaneous EEG recording on 22 schizophrenia patients and 22 gender- and age-matched healthy controls. Source reconstruction and dynamic causal modeling (DCM) analysis were performed to estimate the effective connectivity and casual relationship between frontal and temporal regions before and after habituation.

**Results:** The schizophrenia patients expressed later habituation onset (*p* < 0.01) and hyper-activity in both lateral frontal–temporal cortices than controls (*p* = 0.001). The patients also showed decreased top-down and bottom-up connectivity in bilateral frontal–temporal regions (*p* < 0.01). The contralateral frontal–frontal and temporal–temporal connectivity showed a left to right decreasing (*p* < 0.01) and right to left strengthening (*p* < 0.01).

**Conclusions:** The results give causal evidence for E/I imbalance in schizophrenia during dichotic auditory processing. The altered effective connectivity in frontal–temporal circuit could represent the trait bio-marker of schizophrenia with auditory hallucinations.

## Introduction

Auditory verbal hallucinations (AVHs) are a core symptom of schizophrenia (SCZ), affecting ~60–80% of SCZ patients (1). Auditory verbal hallucinations has been found to be related to the alternation of neural activities (2), such as excitatory–inhibitory (E/I) balance (3). Excitatory–inhibitory balance is the balance between excitatory and inhibitory synapses which operates the information processing in neural circuit (4). This E/I balance has been shown to play an important role in the perceptual representations (5). Hallucination, as a kind of perceptual disorder, is closely related to inhibitory deficiencies (6). However, the casual relationship and mechanisms by which E/I dysfunctions relate to clinical symptoms and cognitive deficits in SCZ patients remain unclear.

Excitatory–inhibitory balance in the neural circuit has been widely studied in the form of habituation (7, 8). Habituation is a fundamental process manifested by a stimulus-specific decrement of neuronal responses, that is, weakening in neural activity to repeated sensory stimulation with the same or a similar stimulus (9). Repetition-related reductions in neural activity have previously been reported at multiple time scales ranging from milliseconds (10), seconds (11), minutes (12), to weeks (13). Furthermore, habituation is considered to protect higher cortical centers from flooding with irrelevant information (14–16), and to protect processing of the first response by filtering redundant sensory inputs (17, 18). Distinctive brain regions such as auditory cortex (19) and frontal sources have been found to be simultaneously active during the course of habituation (10).

Previous works use electroencephalography (EEG) recordings to study habituation to redundant sensory input, or sensory gating, and find a time-dependent decrease of amplitude (20) might account for the habituation of the response. Some scholars believe that the predominantly bottom-up process directed gamma-band (30–50 Hz) influences are controlled by predominantly top-down directed alpha-beta band (8–20 Hz) influences (21), and this phenomena also can be find in attention (22) and auditory perception (23). In addition to this theory, there have been several empirical studies which analyzed event related potential to reveal auditory hallucination was the deficit of sensory gating (24, 25) and inhibitory deficit (26). Therefore, we proposed that auditory habituation was a perceptual process that could reflect the neural network processing deficit of hallucination in SCZ.

The Dichotic Listening Test provides a reliable instrument for the separate measurement of bottom-up and top-down processes (27). During the dichotic listening, participants have to shift their attention to the left ear and inhibit the bottom-up information from the right ear and vice versa, which is believed to represent top-down inhibitory control and adequate monitoring of incoming auditory information (28). Researchers found hallucinatory experiences were associated with enhanced report from the right ear in the forced-right condition which suggested reduced bottom-up and inhibitory top-down control in SCZ (28–30). Interestingly, a meta-analysis of dichotic listening studies has suggested reduced left-hemispheric language lateralization as a trait marker for the occurrence of auditory hallucinations within the SCZ population (30). In addition, a frontal inhibitory neuron activity in affective circuit of rodent was recorded recently (31), which reveals a crucial role of frontal GABAergic interneurons in E/I balance. Therefore, we predicted that in SCZ patients the dichotic listening response would be a reliable paradigm in probing AVHs.

So far there has been very little references made between the altered dichotic listening responses in SCZ and the underlying neural mechanisms, especially in the direction of neural E/I balance. Dynamic causal modeling (DCM) is a useful method in this regard. Dynamic causal modeling is a kind of non-linear algorithm which generate dynamic model to present brain effective connectivity and explain the casual relationship between brain regions (32). This is because DCM can provide information on the mechanisms that represent potential key dimensions of psychiatric disease, e.g., excitation–inhibition balance and synaptic plasticity by N-methyl D-aspartate receptors or its regulation by neuromodulatory transmitters such as dopamine or acetylcholine (33, 34). For example, there is evidence that links gamma- and alpha-band to neurotransmitter systems involving parvalbumin-positive GABAergic interneurons and glutamatergic pyramidal cells (35–37). The relationship between beta-band and dopaminergic system (38) has also been reported. Dynamic causal modeling can explain causal relationship through dynamically interacting sources. Our previous work used DCM analysis and provided the evidence of early drug response-related alterations in effective brain connectivity (39).

In the present study, we used EEG to obtain time-frequency oscillations during the dichotic listening to examine the time difference between SCZ and healthy controls in terms of when the habituation emerges. We also used source reconstruction methods to explore brain areas responsible to the habituation responses, and then applied DCM analysis (32, 40) to estimate the effective connectivity alteration during habituation between these brain areas. Our hypotheses were as follows: (1) Based on previous findings of inhibitory deficit (28), the SCZ patients would have delayed habituation onset; (2) on the basis of source analysis, the regional activities would be different between SCZ patients and healthy controls, both before and after habituation; (3) the connectivity exerted by regional activity would be different between SCZ and healthy control during habituation.

## Materials and Methods

### Participants

Thirty-three patients at their first presentation of psychosis were recruited from psychiatry department of Second Affiliated Hospital, Zhejiang University School of Medicine. Because of the cooperation of SCZ patients, a total of 22 patients completed the whole experiment and got analyzable data. They were diagnosed as having paranoid schizophrenia with auditory hallucinations according to the ICD-10 criteria (41) after a semi-structured clinical interview. The instrument of the interview we used was The Mini-International Neuropsychiatric Interview (42). The patients had experienced their first acute episode with the disease durations from 2.5 to 5.5 months (mean 4.22 ± 1.10) at the time of EEG recording. All the patients were drug naïve. The severity of clinical symptoms was assessed with the positive and negative syndrome scale (PANSS) (43) on the day when EEG was recorded (Table 1). The PANSS P3 score above 3 was considered as the criteria of having hallucinations.

**Table 1:** Mean and standard deviations for demographic characteristics of two group participants and symptom ratings in schizophrenia patients.

	**Schizophrenia** **(*****n*** **= 22)**	**Heath controls** **(*****n*** **= 22)**	**Statistic value**
Age	19.95 (3.73)	21.50 (2.44)	*F*_(1, 42)_ = 2.64, *p* = 0.11
Gender (male/female)	12/10	8/14	χ^2^ = 1.47, *df* = 1, *p* = 0.36
Disease duration (months)	4.22 (1.10)	NA	
Intelligence quotient	101.86 (7.32)	103.18 (7.87)	*F*_(1, 42)_ = 0.08, *p* = 0.78
Educational years	12.95 (2.59)	13.18 (2.78)	*F*_(1, 42)_ = 3.58, *p* = 0.07
PANSS total	67.25 (7.52)	NA	
PANSS positive	19.12 (4.15)	NA	
PANSS negative	10.45 (2.04)	NA	
PANSS general	38.21 (6.28)	NA	
PANSS hallucination (P3)	4.65 (1.10)	NA	

*PANSS, positive and negative syndrome scale; NA, not applicable*.

The healthy controls were 22 age- and gender matched volunteers recruited from medical staff and the local community. There is no difference in educational level and intelligence quotient between SCZ and healthy controls (Table 1). They received a semi-structured clinical interview to exclude any current or lifetime evidence of psychiatric disorder.

The subjects with a history of neurological disorder or substance abuse disorder, were excluded. All participants had no auditory deficit. Before EEG recording, all subjects accepted a 3.0 T magnetic resonance imaging scan to ensure they were free from any brain structural abnormalities.

All participants were right-handed.

### Description of Stimuli

**Figure 1:**
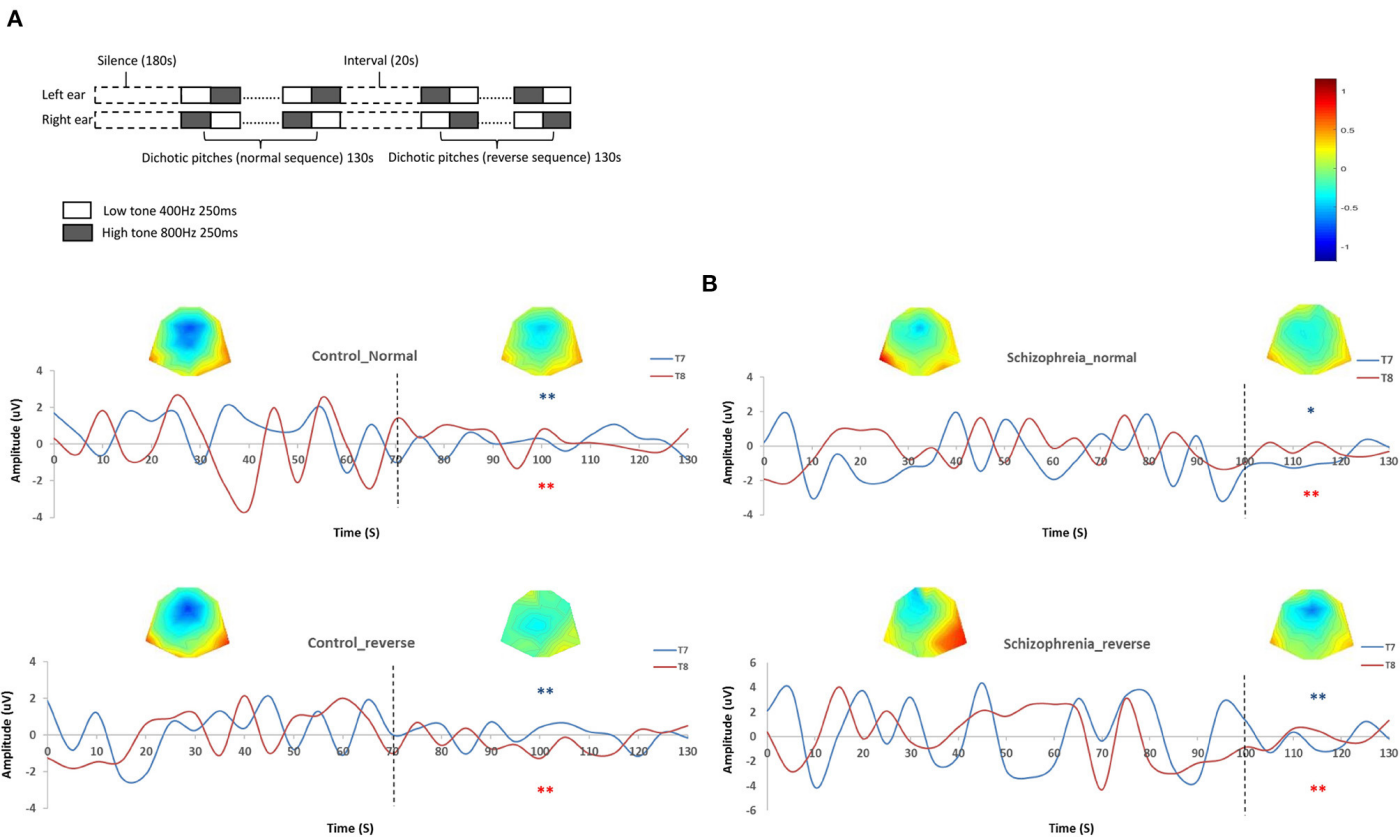
**(A)** Schematic diagram of dichotic listening. **(B)** Habituations during dichotic listening. The left two diagrams are the grand average amplitudes in controls during normal and reverse sequences on T7 and T8 sensors with topographic map. The dot lines mark the onset of habituations (time-point of 70 s in both sequences and sensors). The right two diagrams are the grand average amplitudes in schizophrenia patients during normal and reverse sequences on T7 and T8 sensors with topographic map. The dot lines mark the onset of habituations (time-point of 100 s in both sequences and sensors). *Absolute amplitude value after habituation onset is significant lower than those before habituation onset (*p* < 0.05). **Absolute amplitude value after habituation onset is significantly lower than those before habituation onset (*p* < 0.01).

The stimuli were tones of 70 dB SPL and 250 ms sine waves at equal amplitude and followed each other without pause, which consisted of a 400-Hz and a higher 800 Hz tones. The paired tones switched from one ear to another. When the right ear received the high tone, the left ear received the low one, and vice versa (44). We edited two musical sequences using Adobe Audition 3.0 (Adobe Systems Incorporated, www.adobe.com): the one that began with a 400-Hz tone in the left ear was defined as the normal sequence and the one that began with a 800-Hz tone in the left ear was defined as the reversed sequence. Both sequences lasted for 130 s. Before the auditory task, there was a 5-min silence recording. Between normal and reverse sequence, there was a 20-s interval (Figure 1A).

### EEG Recording

Participants sat in a semi-darkened quiet room with their eyes focused on a white cross on the screen. Electroencephalography recordings were performed with 64 Ag-AgCl scalp electrodes placed according to the International 10/20 system with the impedances below 5 kΩ. The linked mastoids of two sides were attached as the reference electrode. The electrodes placed at the supra-orbitally to the right eye were the bipolar recordings of electro-ocular activity (EOG). Electroencephalography was continuously recorded with a sampling rate of 1,000 Hz.

### Data Analysis

#### Pre-processing

Offline preprocessing was performed using SPM12 (http://www.fil.ion.ucl.ac.uk/spm), running in Matlab R2021a (Mathworks Inc., Natick, MA, USA). Electroencephalography signals were band-pass filtered offline (0.5–50 Hz) with notch-off 50 Hz. Independent component analysis (ICA) and extended infomax algorithm implemented in fieldtrip toolbox (fieldtrip. Fcdonder. NL) are used to eliminate EEG trails including electrooculogram, electrocardiogram, or electromyogram. A sweep in which the EEG exceeded ±70 μV was excluded from analysis.

Spectral estimates were derived in successive temporal windows of 500 ms duration, according to the frequency of five bands: delta (0.5–3.5 Hz), theta (3.5–7.5 Hz), alpha (7.5–12.5 Hz), beta (12.5–30 Hz), and gamma (30–50 Hz) (45). The epochs were extracted from silence, normal, and reverse sequence, respectively. Fifty artifact free 500-ms epochs which followed the “high-low” cycle were averaged for source-reconstruction and further dynamic casual modeling analysis. Furthermore, the habituation onset was set as the 50% shrink of absolute grand averaged amplitude (46), and then the sequences were divided into before and after habituation segments according to the attenuated amplitude in normal and reverse sequences, respectively (Figure 1B).

#### Source-Reconstruction

The source locations of brain electrical activity were using 3D source reconstruction toolbox of SPM 12. In terms of empirical priors, the algorithm transformed the EEG data and automatically selected multiple cortical sources with compact spatial support (47). The specific 3-D models were constructed in the SPM software followed the default approach. We performed coregistration of sensors with SPM's template head model based on the MNI coordinates for each subject. Based on an empirical Bayesian formalism (47–49), the source space was constructed using a canonical cortical mesh, defined in a standard stereotactic space. Based on a spatial normalization transformation (47), we generated each source of the mesh in subject space, which was directly corresponding with a location in MNI space. Followed these steps, the 3D images of each subject were generated for further group comparison.

#### Dynamic Casual Modeling

We use DCM12 as implemented in the SPM12 (50). We extracted average EEG times series for each subnetwork from individual subject source and specified a fully connected DCM model for each participant to compare all possible nested models (51). The model was then estimated using spectral DCM, which fits the complex cross-spectral density to analyze source connectivity before and after habituation (Figure 1B). Then we take the subject-specific DCMs to the second level, where we used parametric empirical Bayes (PEB) routines for group level reasoning inference (51). Specifically, we designed a 2 (group) × 2 (sequence) × 2 (habituation) matrix to examine directional connectivity difference between schizophrenia and healthy controls. Next, we used Bayesian model reduction to test all nested models within each full PEB model and to “prune” connection parameters. The Bayesian model averaging were used to generate parameters of the best 256 pruned models and group estimates of connection parameters. Furthermore, the posterior probability for each model of the log Bayes factor were compared (52). We report effects (connection strengths) as significant with a posterior probability of >0.95.

### Statistical Analysis

We used a standard voxel-wise statistical analysis to identify source activations in each participant independently. We calculated the parameters for all brain voxels, and do contrasts for each group via general linear model. A second level analysis was then conducted to gain the main effect of brain activity between SCZ and healthy control group at each stage and frequency band. Statistical inferences were made at a whole brain corrected cluster level of *p* = 0.001.

The comparison of absolute amplitude value between two groups was performed using *t*-test. The DCM parameters were contrasted using repeated measure variance analysis between two groups during difference stages, and a *post-hoc t*-test with Bonfferoni correction was performed when group effect was significant. Before comparison, a normality test was conducted and for all the comparisons, the *p*-value < 0.05 was considered to be significance.

## Results

### Habituation

We chose T7 and T8 as the representative auditory electrodes (53), and generated the grand averaged amplitudes in both groups. The absolute amplitudes of T7/T8 were used for comparing difference between two groups. Both in normal and reverse dichotic pitch sequence, the amplitude attenuated after 70 s in healthy participants [normal sequence: *t*_(42)_ = 4.62/2.94, *p* = 0.00/0.01; reverse sequence: *t*_(42)_ = 4.11/4.05, *P* = 0.00/0.00], while in SCZ the time point is 100 s [normal sequence: *t*_(42)_ = 2.25/2.67, *p* = 0.04/0.01; reverse sequence: *t*_(42)_ = 3.73/2.78, *P* = 0.00/0.01] (Figure 1B).

### Regional Activity

**Figure 2:**
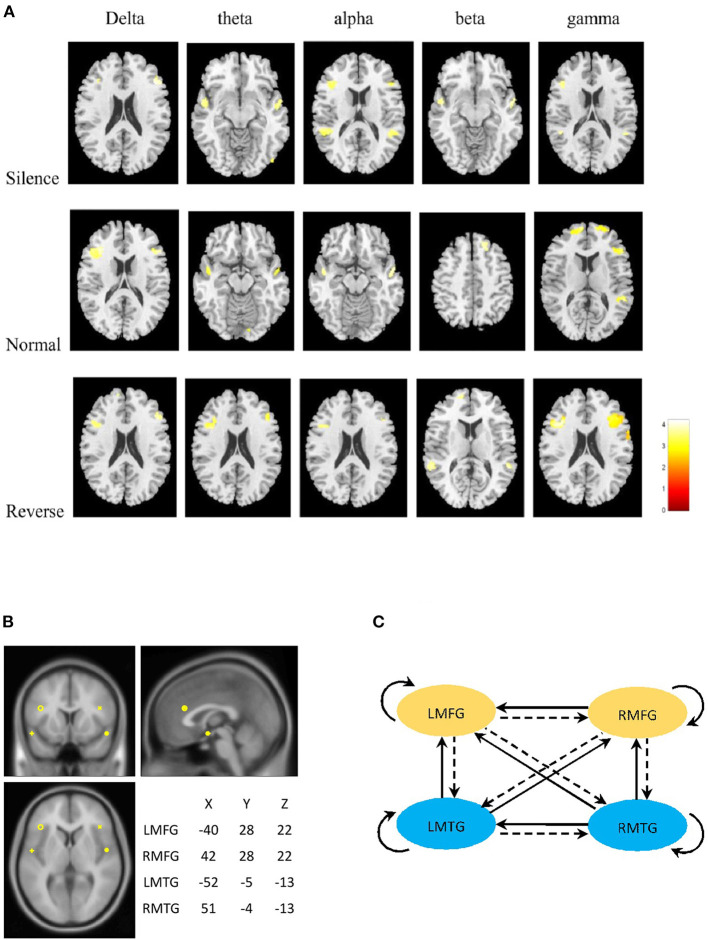
**(A)** Statistical parametric maps of the two groups. The column panels represent the five frequency bands (delta, theta, alpha, beta, and gamma). The row panels represent the three stages (silence, normal, and reverse). Each image presents the comparison between Schizophrenia and Healthy controls. The detailed information is presented in Table 2. The figures were generated using xjView (http://www.alivelearn.net/xjview). **(B)** Prior locations of the nodes in the models. Activity sources were modeled as equivalent dipoles. Their prior mean locations in mm are superimposed over an MRI of a standard brain in MNI space. **(C)** Fully connected model for DCM analysis. LMFG, left middle frontal gyrus; RMFG, right middle frontal gyrus; LMTG, left middle temporal gyrus; RMTG, right middle temporal gyrus.

In all frequency bands, the common brain areas with significant difference between SCZ and healthy controls were bilateral middle frontal and middle temporal gyrus during three stages (silence, normal sequence, reverse sequence). Group comparison revealed higher brain activities in SCZ patients than those in healthy controls in left/right middle frontal gyrus (L/RMFG) and left/right middle temporal gyrus (L/RMTG) cross five frequency bands during all stages. There were higher brain activities of alpha band in SCZ patients than those in healthy controls in left/right superior temporal gyrus during silence stage. Also, there were higher brain activities of gamma band in SCZ patients than those in healthy controls in left/right superior frontal gyrus and right superior temporal gyrus during normal sequence stage (Figure 2A; Table 2).

**Table 2:** Coordinates of remarkable differences in sources between schizophrenia patients and healthy controls on five bands during four stages.

	**Delta (0.5–3.5 Hz)**	**Theta (3.5–7.5 Hz)**	**Alpha (7.5–12.5 Hz)**	**Beta (12.5–30 Hz)**	**Gamma (30–50 Hz)**
Silence	LMFG (−44, 32, 22)	LMTG (−50, −3, −14)	LMFG (−40, 32, 19)	LMTG (−49, −2, −14)	LMFG (−48, 24, 18)
	RMFG (46, 28, 22)	RMTG (53, −3, −14)	LSTG (−57, −42, 13)	RMTG (52, −4, −13)	
			RSTG (52, −42, 13)		
Normal	LMFG (−40, 29, 24)	LMTG (−51, −5, −14)	LMTG (−51, −3, −14)	RMFG (16, 35, 48)	LSFG (−20, 59, 12)
	RMFG (45, 28, 24)	RMTG (51, −2, −14)	RMTG (53, −3, −14)		RSFG (18, 62, 12)
					RMFG (45, 30, 18)
					RSTG (53, −42, 12)
Reverse	LMFG (−42, 27, 22)	LMFG (−48, 28, 24)	LMFG (−48, 20, 22)	LSTG (53, −42, 12)	RMFG (43, 31, 22)
	RMFG (46, 28, 22)	RMFG (48, 30, 22)		RSTG (−57, −40, 12)	LMFG (−43, 25, 22)

*LMFG, left middle frontal gyrus; RMFG, right middle frontal gyrus; LMTG, left middle temporal gyrus; RMTG, right middle temporal gyrus; LSTG, left superior temporal gyrus. Only significant results are presented, t_(42)_ = 3.30, p = 0.001*.

From the statistical differences, we determined current dipole with a prior variance of 12 mm as LMFG [−40 28 22], RMFG [42 28 22], LMTG [−52 −5 −13], and RMTG [51 −4 −13] that were in the center of a radius of the group maxima difference (Figure 2B).

### Effective Connectivity

**Figure 3:**
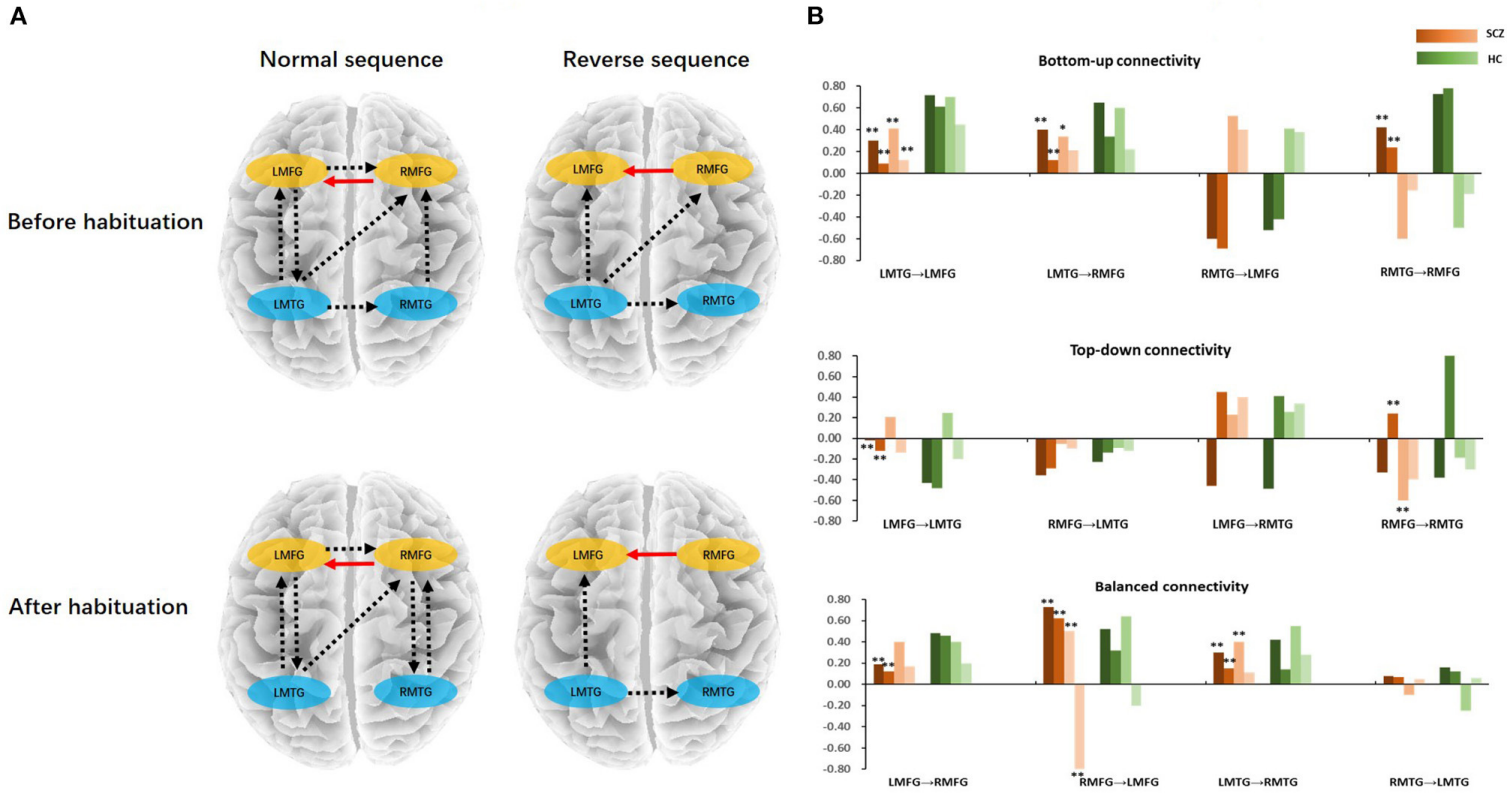
**(A)** The connectivity differences between schizophrenia and healthy controls in normal/reverse sequence before/after habituation. **(B)** Bottom-up, top-down, and balanced connectivity difference between schizophrenia and healthy controls. In the histogram, the red bars represent the schizophrenia; the green bars represent the healthy controls; the color bars from deep to shallow represent normal sequence before habituation, normal sequence after habituation, reverse sequence before habituation, and reverse sequence after habituation, respectively. *Schizophrenia vs. healthy controls (*p* < 0.05), **Schizophrenia vs. healthy controls (*p* < 0.01). LMFG, left middle frontal gyrus; RMFG, right middle frontal gyrus; LMTG, left middle temporal gyrus; RMTG, right middle temporal gyrus; SCZ, schizophrenia; HC, healthy control.

Finally, we tested with generative modeling how habituation differentially affected forward and backward connections among sources between two groups. The nested full models for DCM were showed in Figure 2C. We used spectral DCM to characterize neuronal dynamics of local and long-range connections. Figure 3A shows the group difference of four stages between schizophrenia and healthy controls after PEB procedure. We found lower bottom-up and top-down connectivity in bilateral frontal–temporal loop during both sequences in schizophrenia than those in controls. Meanwhile, we found an unbalanced architecture of reciprocal connections between bilateral frontal (strengthened RMFG to LMFG; decreased LMFG to RMFG) as well as temporal regions (decreased LMTG to RMTG) in schizophrenia. Figure 3B shows the group average parameters of each directional connectivity before and after habituation. In the bilateral frontal–temporal loop, the top-down frontal–temporal connections were lower in schizophrenia than those in healthy controls during dichotic stimuli [LMFG to LMTG: *F*_(1, 42)_ = 4.96, *p* = 0.03. RMFG to RMTG: *F*_(1, 42)_ =5.03, *p* = 0.03]. The distinct change in bottom-up connection is “LMTG to LMFG” and “LMTG to RMFG,” which decreased in schizophrenia patients both in normal and reverse sequence, especially in the normal sequence after habituation [*F*_(1, 42)_ =19.94, *p* = 0.00; *F*_(1, 42)_ =9.94, *p* = 0.01]. The bilateral frontal–frontal and temporal–temporal connections showed imbalance during dichotic stimulation, which presented the weakening of left to right [LMFG to RMFG: *F*_(1, 42)_ = 5.07, *p* = 0.03; LMTG to RMTG: *F*_(1, 42)_ = 5.97, *p* = 0.02] and the increasing of right to left connectivity [RMFG to LMFG: *F*_(1, 42)_ = 18.93, *p* = 0.00] (Table 3).

**Table 3:** BMA parameters generated from nested PEB models for all connections in schizophrenia and healthy participants before and after habituation.

	**Schizophrenia (*****n*** **=** **22)**				**Healthy control (*****n*** **=** **22)**				**Group effect**		***Post-hoc***
	**Normal sequence**		**Reverse sequence**		**Normal sequence**		**Reverse sequence**		***F***_**(1, 42)**_	***p***	
	**① Before habituation**	**② After habituation**	**③ Before habituation**	**④ After habituation**	**⑤ Before habituation**	**⑥ After habituation**	**⑦ Before habituation**	**⑧ After habituation**			
*Bottom-Up Mean (S.E.M)*											
**LMTG** **→** **LMFG**	0.30 (0.04)	0.09 (0.05)	0.41 (0.12)	0.12 (0.05)	0.72 (0.27)	0.61 (0.23)	0.70 (0.08)	0.45 (0.38)	19.94	0.00	① vs. ⑤, ② vs. ⑥, ③ vs. ⑦, ④ vs. ⑧*p* <0.01
**LMTG** **→** **RMFG**	0.40 (0.12)	0.12 (0.06)	0.34 (0.28)	0.21 (0.12)	0.65 (0.24)	0.34 (0.12)	0.60 (0.23)	0.22 (0.16)	9.94	0.01	① vs. ⑤, ② vs. ⑥*p*<*p* <0.01; ③ vs. ⑦*p* <0.05
RMTG → LMFG	−0.60 (0.33)	−0.69 (0.25)	0.53 (0.41)	0.40 (0.23)	−0.52 (0.31)	−0.42 (0.21)	0.41 (0.25)	0.38 (0.22)	0.67	0.42	
**RMTG** **→** **RMFG**	0.42 (0.16)	0.24 (0.12)	−0.60 (0.35)	−0.16 (0.09)	0.73 (0.23)	0.78 (0.13)	−0.50 (0.23)	−0.19 (0.09)	6.03	0.02	① vs. ⑤, ② vs. ⑥*p* <0.01
*Top-Down Mean (S.E.M)*											
**LMFG → LMTG**	−0.02 (0.02)	−0.12 (0.07)	0.21 (0.15)	−0.14 (0.04)	−0.43 (0.13)	−0.48 (0.22)	0.25 (0.12)	−0.20 (0.08)	4.96	0.03	① vs. ⑤, ② vs. ⑥*p* <0.01
LMFG → RMTG	−0.46 (0.22)	0.45 (0.21)	0.23 (0.17)	0.40 (0.19)	−0.49 (0.28)	0.41 (0.19)	0.26 (0.17)	0.34 (0.18)	0.61	0.44	
RMFG → LMTG	−0.36 (0.11)	−0.29 (0.13)	−0.05 (0.03)	−0.10 (0.05)	−0.23 (0.09)	−0.14 (0.06)	−0.09 (0.06)	−0.12 (0.04)	1.41	0.24	
**RMFG** **→** **RMTG**	–0.33 (0.12)	0.24 (0.18)	–0.60 (0.25)	–0.40 (0.29)	–0.38 (0.16)	0.80 (0.42)	–0.19 (0.13)	–0.30 (0.17)	5.03	0.03	② vs. ⑥, ③ vs. ⑦*p* <0.01
*Balanced Mean (S.E.M)*											
**LMFG** **→** **RMFG**	0.19 (0.08)	0.12 (0.06)	0.40 (0.21)	0.17 (0.08)	0.48 (0.26)	0.46 (0.23)	0.40 (0.18)	0.20 (0.12)	5.07	0.03	① vs. ⑤, ② vs. ⑥*p* <0.01
**LMTG** **→** **RMTG**	0.30 (0.12)	0.15 (0.07)	0.40 (0.21)	0.11 (0.06)	0.42 (0.25)	0.14 (0.08)	0.55 (0.23)	0.28 (0.13)	5.97	0.02	① vs. ⑤, ③ vs. ⑧, ⑥ vs. ⑨*p* <0.01
**RMFG** **→** **LMFG**	0.73 (0.14)	0.62 (0.23)	0.50 (0.14)	–0.80 (0.12)	0.52 (0.20)	0.32 (0.15)	0.64 (0.22)	–0.20 (0.11)	18.93	0.00	① vs. ⑤, ② vs. ⑥, ③ vs. ⑦, ④ vs. ⑧*p* <0.01
RMTG → LMTG	0.08 (0.02)	0.07 (0.03)	–0.10 (0.05)	0.05 (0.03)	0.16 (0.06)	0.12 (0.08)	−0.25 (0.11)	0.06 (0.04)	1.45	0.23	

*LMFG, left middle frontal gyrus; RMFG, right middle frontal gyrus; LMTG, left middle temporal gyrus; RMTG, right middle temporal gyrus; BMA, Bayesian model averaging; PEB, parametric empirical Bayes*.

## Discussion

In accord with previous work (20), we found a time-dependent decrease of amplitude account for the habituation during the dichotic listening. A seminal work (19) demonstrates that superposition of varying phasic events, can possibly reflect neural habituation and other adaptation processes. In the present study, the SCZ patients showed later habituation onset (100 vs. 70 s) compared to the controls, which implied the patients had protective mechanism deficit in processing flooding information and redundant sensory inputs (14–17). During dichotic listening, the patients had different regional activity compared to the controls in both lateral middle frontal and temporal areas, which indicated the key nodes of neuro-circuit we should pick to explore the connectivity alteration during dichotic listening.

We propose if neuronal communication depends on neuronal synchronization, then dynamic changes in synchronization can flexibly alter the pattern of communication. Habituation to redundant sensory input or sensory gating is a protective mechanism (54) and therefore has been linked to synaptic depression (55). During the dichotic listening, the attention shifts circularly from the left ear to the right ear, which represents top-down inhibitory control and adequate monitoring of incoming auditory information (28). The habituation during dichotic listening may reflect the mechanism of top-down inhibitory control and bottom-up feedforward processing. In the present study, the SCZ patients expressed higher regional activity than controls in bilateral frontal and temporal regions cross five frequency band both during silence and dichotic listening, which may indicate the regional inhibitory deficit in SCZ. The hallucination is known as imbalance between excitatory and inhibitory (6). The result of the present study found the SCZ patients appeared hyper-activity both at silence and task related state in the frontal–temporal areas, which was in line with the view of previous study (6). Further DCM analysis revealed the casual relationship of E/I dysfunctions at the neural circuit level during the habituation of dichotic listening.

To investigate the effective frontotemporal connectivity before and after habituation, we used PEB and BMA to generate the parameters of the best prune models. The result indicated bilateral frontal–temporal circuit played an important role in habituation processing (Figure 3). During habituation, the ipsilateral/contralateral frontal–temporal connections was lower in schizophrenia than those in healthy controls. This results revealed decreased synchrony between frontal and auditory cortices in hallucinating individuals (56). The decreased bottom-up and top-down connectivity with a hyper-local activity revealed an E/I imbalance in schizophrenia during auditory information processing. In regard with the bilateral frontal–frontal and temporal–temporal communication, schizophrenia patients also presented an imbalance connectivity, which showed a decreased left (LMFG/LMTG) to right (RMFG/RMTG) and an increased right (RMFG) to left (LMFG) connections. One possible interpretation of the hyperactivity in frontal and temporal lobe in SCZ patients is that the inhibitory neuron deficits in both the synthesis and reuptake of GABA in these regions in SCZ (57). Previous work has also demonstrated that the inhibitory neurons are thought to create the discrete temporal structure that is necessary for enabling pyramidal neurons to perform specific functions such as the auditory attention switching (58, 59). This kind of deficit weakens the bottom-up and top-down information transmit (60). In the present study, the strength of both the contralateral bottom-up and top-down connectivity was reduced in SCZ, which provided evidence for the GABAergic neuron deficit (61). Different subtypes of inhibitory interneurons could effectively control cortical network activity via feedforward, feedback inhibition, and/or disinhibitory mechanisms (62–64). Our results thus suggested that altered effective connectivity in the frontal–temporal circuit may represent the inhibitory neuron activities deficit at a systematic level, e.g., the weakened feedforward LMTG to LMFG connection and the feedback LMFG to LMTG connectivity, which represented a gating deficit and the excitation/inhibition imbalance (65). Because the SCZ patients in the present study were all at the first episode of the disease, the compensatory mechanism was still online, which may be shown as the increased right to left frontal connection. In other word, the increasing local activity and the reduced top-down/bottom-up connectivity manifested an excitation/inhabitation imbalance in the frontal–temporal circuit. As the time goes by, the imbalance could be cured or even worsen, which remind us that the alteration of effective connectivity in frontal–temporal circuit during habituation may serve as the bio-mark to predict the prognosis of hallucination in SCZ.

There are two limitations in our study. One is the small sample size, the other is that we only carried out a cross-section experiment. A cohort and follow up observation should be performed to observe therapeutic effect via the habituation related effective connectivity in frontal–temporal circuit.

In summary, using EEG and DCM, we found evidence of causal relationship in frontotemporal circuit during habituation of dichotic listening in SCZ. The altered effective connectivity in frontal–temporal circuit could represent the trait bio-marker of schizophrenia with auditory hallucinations. Furthermore, the loop mechanism provided by our study may provide clues and references for future clinical treatment, such as transcranial magnetic stimulation or other physical therapies.

## Data Availability Statement

The raw data supporting the conclusions of this article will be made available by the authors, without undue reservation.

## Ethics Statement

The studies involving human participants were reviewed and approved by Ethics Committee of Second Affiliated Hospital of Zhejiang University School of Medicine. Written informed consent to participate in this study was provided by the participants' legal guardian/next of kin. Written informed consent was obtained from the individual(s), and minor(s)' legal guardian/next of kin, for the publication of any potentially identifiable images or data included in this article.

## Author Contributions

The current research was designed by LZ, BG, and ZL. The data was analyzed by LZ, WY, and XH. The draft was written by LZ, LY, LC, HL, and SY. All authors contributed to the discussion of the results and have approved the final manuscript to be published.

## Funding

This work was supported by the Natural Science Foundation of Zhejiang Province in China (No. LY16H090009) and the Natural Science Foundation of Zhejiang Province in China (No. LQ19H090020). No sponsor played a role in this study.

## Conflict of Interest

The authors declare that the research was conducted in the absence of any commercial or financial relationships that could be construed as a potential conflict of interest.

## Publisher's Note

All claims expressed in this article are solely those of the authors and do not necessarily represent those of their affiliated organizations, or those of the publisher, the editors and the reviewers. Any product that may be evaluated in this article, or claim that may be made by its manufacturer, is not guaranteed or endorsed by the publisher.
